# Cutaneous Metastases of the Synchronous Primary Endometrial and Bilateral Ovarian Cancer: An Infrequent Presentation and Literature Review

**DOI:** 10.1155/2016/4568653

**Published:** 2016-08-11

**Authors:** Gul Kanyilmaz, Meryem Aktan, Mehmet Koc, Siddika Findik

**Affiliations:** ^1^Department of Radiation Oncology, Meram Medicine School, Necmettin Erbakan University, Akyokus Mevkii, 42090 Konya, Turkey; ^2^Department of Pathology, Meram Medicine School, Necmettin Erbakan University, Akyokus Mevkii, 42090 Konya, Turkey

## Abstract

There are limited data about the cutaneous metastases of gynecological malignancies in the literature. Based on this limited number of studies, cutaneous metastases from gynecological malignancies are uncommon occurrences. Cutaneous metastases from the synchronous endometrioid carcinoma of the uterine corpus and bilateral ovaries arising from endometriosis are extremely rare. Herein, we report a 51-year-old woman with FIGO Stage 1A Grade 1 endometrial endometrioid-type adenocarcinoma and synchronous bilateral Stage 1B ovarian endometrioid-type adenocarcinoma who presented 34 months following total abdominal hysterectomy and bilateral salpingo-oophorectomy with skin metastases. After the patient underwent an excisional biopsy, we applied a palliative radiotherapy. The patient received the combination therapy with cisplatin and doxorubicin after the completion of radiotherapy but the disease evolution was rapidly fatal and the patient died 4 months after her admission to our department due to widely disseminated disease.

## 1. Introduction

Metastases to the skin from internal carcinoma are relatively rare, with a reported incidence of 0.7–10% [1]. Cutaneous metastases may be the first presentation of such malignancies, may accompany other symptoms, or may occur during follow-up. As such, breast cancer (23.9%) is the most common origin of cutaneous metastases in women and lung cancer (1.7 to 3.1) is the most common origin in men [2–4]. In the tumors arising from female genital tract, excluding vulvar carcinoma, which is mainly cutaneous, the prevalence of metastases to skin has been cited as 0.7%–1.3% in cervical carcinoma [5], 0.8% in endometrial carcinoma [6], and 1.9% to 5.1% in ovarian carcinoma [7].

Synchronous primary endometrial and ovarian cancer arising from endometriosis is frequently encountered in daily clinical settings. The presence of simultaneous carcinomas involving both the ovary and uterine corpus represents a diagnostic challenge, particularly if the tumors have a similar histology. The classification of these lesions as either two separate primary tumors or as a single primary tumor with a metastasis has significant implications with respect to patient prognosis and recommendations for therapy. Although several morphologic criteria have been proposed as guidelines for the classification of these lesions, certain cases remain difficult to classify with accuracy.

In this paper, we report a 51-year-old woman with FIGO Stage 1A Grade 1 endometrial endometrioid-type adenocarcinoma and synchronous FIGO Stage 1B bilateral ovarian endometrioid-type adenocarcinoma who presented 34 months following total abdominal hysterectomy and bilateral salpingo-oophorectomy with skin metastases. Similar cases in the literature are reviewed.

## 2. Case Presentation

A 51-year-old female, gravida 1 para 1, postmenopausal for 6 years, presented to her gynecologist with a new complaint of vaginal bleeding for 1 month. A systematic physical examination was negative. The patient had no significant past medical history and was not on medication. Since menopause, she had no previous episode of postmenopausal bleeding. Endometrial biopsy and endocervical curettage were performed. The pathologic report was the following: well-differentiated endometrioid-type endometrial adenocarcinoma.

The patient was referred to the gynecologic oncology service and underwent an exploratory laparotomy, total abdominal hysterectomy, bilateral salpingo-oophorectomy, and pelvic and paraaortic lymph node sampling. Surgicopathologic assessment revealed FIGO Stage IA Grade 1 endometrial endometrioid-type adenocarcinoma and synchronous FIGO Stage 1B bilateral ovarian endometrioid-type adenocarcinoma arising from endometriosis. The patient received paclitaxel/carboplatin combination chemotherapy for 6 cycles after surgery. Then she received external pelvic irradiation (4500 cGy) in 25 fractions followed by intracavitary radiation therapy (3500 cGy).

**Figure 1:**
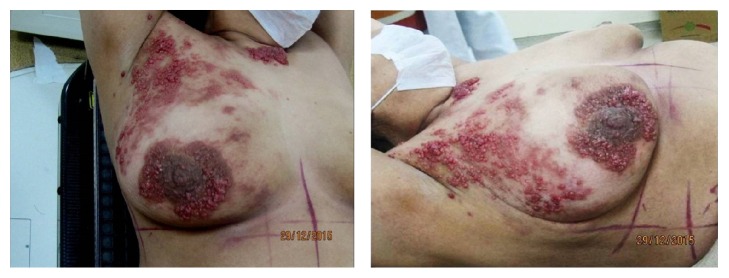
Multiple subcutaneous nodules on the right chest wall.

**Figure 2:**
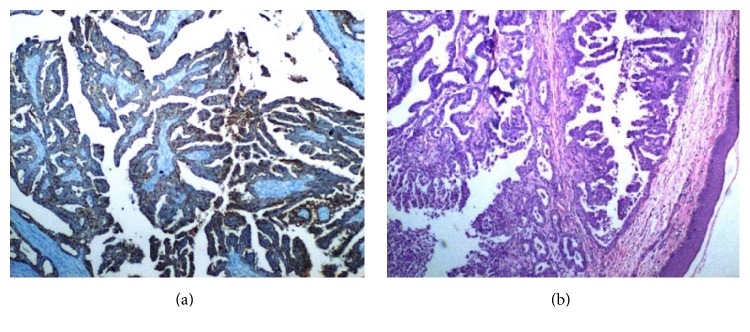
(a) Skin lesion demonstrating subcutaneous tissue with endometrial adenocarcinoma containing glandular structures. (b) Positive immunohistochemical expression with LMW-CK (low molecular weight cytokeratin).

She was followed regularly with a physical examination every 3 months and a chest X-ray every 6 months and was clinically without evidence of disease. Twenty-two months after her initial therapy, scalene lymph node metastasis was seen and confirmed by biopsy. The disease continued to progress while the second-line chemotherapy was applied to the patient. She was referred to our department thirty months after her initial therapy with supraclavicular mass and cutaneous lesions on chest wall skin. Clinical examination revealed the presence of a right supraclavicular lymph node and cutaneous papulobullous lesions located on the right chest wall skin (Figure 1). A cervico-thoraco-abdomino-pelvic computed tomography scan was performed and an abnormally growing supraclavicular lymph node was shown at about 5 cm on the right. Before starting radiotherapy, the patient underwent an excisional biopsy. We report the result of a skin biopsy that disclosed groups of metastatic adenocarcinoma cells in the dermis and subcutaneous tissue (Figures 2(a) and 2(b)). Histopathologic examination demonstrated that the cutaneous metastasis was of genital origin (endometrioid-type adenocarcinoma). Before starting radiotherapy, patient's head was immobilized with a thermoplastic mask before CT simulation. The whole scalene and supraclavicular lymph nodes which were involved by tumor and tumor bed of skin metastasis were included in the Clinical Target Volume (CTV). Three-dimensional conformal radiotherapy (3D-CRT) was used to deliver specified doses to the Planning Target Volume (PTV). The dose was prescribed such that >95% of the PTV received 100% of the prescribed dose. RT delivered 30 Gy at 3 Gy per fraction by 3D-CRT. The patient received the combination therapy with cisplatin and doxorubicin after the completion of radiotherapy but the disease evolution was rapidly fatal and the patient died 4 month after her admission to our department due to widely disseminated disease.

## 3. Discussion

Cutaneous metastasis is a relatively uncommon manifestation of internal malignancies. It most often occurs late in the course of disease but may also be the first presentation of underlying cancers. The overall incidence of cutaneous metastases from visceral neoplasm is 5.3%, ranging from 0.7% to 10% [1, 3]. The tumor with the highest incidence of cutaneous metastases is breast cancer. Lung cancer, colorectal cancer, renal cancer, ovarian cancer, and bladder cancer all have similar rates for cutaneous metastases between 3.4% and 4% [3]. In the tumors arising from female genital tract, the prevalence of metastases to skin has been cited as 0.7%–1.3% in cervical carcinoma [5], 0.8% in endometrial carcinoma [6], and 1.9% to 5.1% in ovarian carcinoma [7]. In the literature, we did not find such a study that reported cutaneous metastases from the endometrioid-type endometrial adenocarcinoma of uterine corpus and synchronous bilateral ovarian endometrioid-type adenocarcinoma.

Histopathological appearance is the most important feature in the diagnosis of cutaneous metastases, as it is similar to that of the primary tumor. In our study, histology of the sections showed pigmented skin overlying metastatic malignant tumors consistent with endometrioid-type adenocarcinoma from the endometrial and the synchronous bilateral ovarian cancer. Because of advances in cancer therapy, patients with cutaneous metastases may live longer than before. Nonetheless, cutaneous metastases are still a poor prognostic sign, especially in patients with cancer of the lung, ovary, upper respiratory tract, or upper digestive tract [8]. Most cases of cutaneous metastases are indicative of widespread dissemination of the disease and death with poor prognosis; the overall survival time from appearance of skin metastases has been cited as 4–12 months [9, 10]. The factor that affects the survival is the time elapsed between diagnosis and the occurrence of the cutaneous metastases [7]. The treatment for most patients is palliative, and although chemotherapy and radiotherapy are often used in these patients, they are ineffective in many cases [11]. Our patient underwent an excisional biopsy before the starting of the radiation therapy and received 30 Gy at 3 Gy per fraction by 3D-CRT to whole scalene and supraclavicular lymph nodes which were involved by tumor and tumor bed of skin metastases. The patient received the combination therapy with cisplatin and doxorubicin after the completion of radiotherapy but the disease evolution was rapidly fatal and the patient died 4 month after her admission to our department due to widely disseminated disease.

## 4. Conclusions

Synchronous primary endometrial and ovarian cancer is frequently encountered in daily clinical settings but cutaneous metastases following synchronous primary endometrial and ovarian cancer are extremely rare. This study is the first one that reported the cutaneous metastases from the synchronous endometrioid carcinoma of the uterine corpus and ovary arising from endometriosis. It is a clinical sign of a widely disseminated disease with a poor prognosis: once cutaneous lesions are diagnosed, survival approximates 4–6 months.
